# Spatial and Temporal Variation of Drought Based on Satellite Derived Vegetation Condition Index in Nepal from 1982–2015

**DOI:** 10.3390/s19020430

**Published:** 2019-01-21

**Authors:** Binod Baniya, Qiuhong Tang, Ximeng Xu, Gebremedhin Gebremeskel Haile, Gyan Chhipi-Shrestha

**Affiliations:** 1 Key Laboratory of Water Cycle and Related Land Surface Processes, Institute of Geographic Sciences and Natural Resources Research, Chinese Academy of Sciences, Beijing 100101, China; baniya@igsnrr.ac.cn (B.B.); xuxm@igsnrr.ac.cn (X.X.); gg.haile@igsnrr.ac.cn (G.G.H.); 2 University of Chinese Academy of Sciences, Beijing 100049, China; 3 Department of Environmental Science, Patan Multiple Campus, Tribhuvan University, Kathmandu 44613, Nepal; 4 École Supérieure d’Amenagement du Territoire, Université Laval, 1628 Pavillon Savard, Université Laval, Québec City, QC G1K7P4, Canada; gyan.chhipi@alumni.ubc.ca

**Keywords:** VCI, drought, exceedance probability, climate, Nepal

## Abstract

Identification of drought is essential for many environmental and agricultural applications. To further understand drought, this study presented spatial and temporal variations of drought based on satellite derived Vegetation Condition Index (VCI) on annual (Jan–Dec), seasonal monsoon (Jun–Nov) and pre-monsoon (Mar–May) scales from 1982–2015 in Nepal. The Vegetation Condition Index (VCI) obtained from NOAA, AVHRR (National Oceanic and Atmospheric Administration, Advanced Very High Resolution Radiometer) and climate data from meteorological stations were used. VCI was used to grade the drought, and the Mann–Kendall test and linear trend analysis were conducted to examine drought trends and the Pearson correlation between VCI and climatic factors (i.e., temperature and precipitation) was also acquired. The results identified that severe drought was identified in 1982, 1984, 1985 and 2000 on all time scales. However, VCI has increased at the rate of 1.14 yr^−1^ (*p* = 0.04), 1.31 yr^−1^ (*p* = 0.03) and 0.77 yr^−1^ (*p* = 0.77) on the annual, seasonal monsoon and pre-monsoon scales, respectively. These increased VCIs indicated decreases in drought. However, spatially, increased trends of drought were also found in some regions in Nepal. For instance, northern areas mainly in the Trans-Himalayan regions identified severe drought. The foothills and the lowlands of Terai (southern Nepal) experienced normal VCI, i.e., no drought. Similarly, the Anomaly Vegetation Condition Index (AVCI) was mostly negative before 2000 which indicated deficient soil moisture. The exceedance probability analysis results on the annual time scale showed that there was a 20% chance of occurring severe drought (VCI ≤ 35%) and a 35% chance of occurring normal drought (35% ≤ VCI ≤ 50%) in Nepal. Drought was also linked with climates in which temperature on the annual and seasonal monsoon scales was significant and positively correlated with VCI. Drought occurrence and trends in Nepal need to be further studied for comprehensive information and understanding.

## 1. Introduction

Drought is a serious phenomenon and ranks first among all natural hazards in terms of the number of people affected globally [1]. Drought hazards increase slowly, which often accumulate over a considerable period and may linger for years after termination [2]. It has been recognized as one of the sensitive environmental disasters affecting natural ecosystem, agriculture and hydrological systems [3]. As the onset and end of drought are difficult to determine, the drought severity is sometimes incredibly difficult to quantify. Droughts appear as a condition of below average rainfall and slowly develop as an extreme climatic event that has severe impacts on the environment [4]. Nearly, half of the countries in the world have suffered from drought [5,6]. According to a recent Intergovernmental Panel on Climate Change (IPCC) study, production of rice, maize and wheat in the past few decades have declined in many parts of Asia due to increasing water stress, temperature, frequency of ELNino events and reduction in the number of rainy days [7]. The occurrence of severe drought in many parts of Northern China has caused large economic losses [8]. Similarly, India is one of the most vulnerable and drought prone countries where drought has been reported at least once in every three years in the last five decades [9,10]. Nepal lies in the same hemisphere with China and India and is affected by the south Asian monsoon and global climate changes. Therefore, monitoring and early warning of drought in Nepal are required on the local and regional scales. 

Nepal experienced drought in 1972, 1977, 1982 and 1992 and also frequent dry spells since 2002 (i.e., from 2004 to 2006 during both the dry and wet monsoon) [11]. Nepal droughts over the last 33 years had moderate to extreme drought intensity in Nepal [12]. Drought often occurred in mid and far western Nepal [13], especially during the winter and summer season [14]. The worst widespread droughts in central Nepal were recorded in 2004, 2005, 2006, 2009 in summer and 2006, 2008 and 2009 in winter [15]. Tree rings also revealed an intensified spring drought [16] and spring and summer drought occurred in the Trans-Himalayan regions in Nepal [17]. Droughts are more common in the lowlands and in the western hills of Nepal [11,18]. The hilly and mountain districts in Far and Midwestern parts of Nepal are drought prone regions [19]. Similarly, Trans-Himalayan regions experienced prolonged drought [17] and extremely dry conditions throughout the year [11]. Drought in Nepal is due to the country’s high climatic variation with volatile temperature and precipitation patterns in the elevation ranging from the low altitudinal zone at 60 m in the south to a high altitude at 8848 m above sea level in the north. About 80% of the total annual precipitation occurs during summer [20] while the winter precipitation only contributes 3% of total annual precipitation [21]. The rainfall distribution patterns are erratic and spatially varied due to an orographic effects and influences of the Asian monsoon which cause southern and eastern parts to receive more rainfall while northern and western parts receive less [22]. The considerable temperature rise in Nepal is 1.5 °C at the rate of 0.06 °C·yr^−1^ from 1972 to 1994 [23], 0.04 °C·yr^−1^ from 1975 to 2007 [24] and 0.03 °C·yr^−1^ from 1982 to 2015 [25]. The temperature has significantly increased across the country, but pre-monsoon precipitation showed significant decreases in the High-Himalayan regions where very wet days and extremely wet days decreased significantly [26]. These increased temperature and decreased precipitation patterns showed a high risk of drought. In recent years, drought has also emerged as a source of vulnerability in rainfed agriculture in Nepal [14,27,28]. 

In order to minimize further risk and potential hazards, it is necessary to monitor and assess drought using scientific tools. Traditional drought monitoring methods mostly rely on ground based observation of meteorological and hydrological data such as temperature, precipitation, soil moisture, evapotranspiration and surface runoff. Based on this point data, several drought indices have been derived in recent decades such as the standardized precipitation index [4], the Palmer drought severity index [29], crop moisture index [30], soil moisture drought index [31] and crop-specific drought index [32]. Besides these indices, indices by Penman, Thornthwaite [33,34,35] and Keetch [36] have been used in limited cases [37]. When applying these methods, data points are interpolated which makes it difficult to ensure reliability because of the limited spatial density and distribution of the observation stations [38,39]. Remote sensing is an advanced and useful technology for monitoring drought. There were many drought indices constructed based on remote sensing of vegetation such as the normalized difference vegetation index (NDVI) [40], temperature vegetation drought index (TVDI) [41] and vegetation condition index (VCI) [42]. The sensitivity of VCI for drought monitoring is significantly higher than NDVI and TVDI [43] which can reduce the impacts of geographical location, ecological system and soil condition [42,44]. VCI is also a better indicator of moisture deficiency than NDVI because it separates climate signals from the long-term ecological signals [45]. Therefore, VCI can be used in non-homogenous areas to monitor and analyze drought more accurately than other remote sensing based indices. As a result, VCI has been widely used in drought monitoring and analysis [46,47,48,49] and many studies have verified its reliability. In Nepal, previous studies have focused on spatial and temporal analysis of drought derived from the Standardized Precipitation Index (SPI) and linked with climate indices [12,13,14] through the interpolation of point data. Drought risk assessment with temporal and spatial analysis using SPI has been carried out in central Nepal [15]. In addition, tree ring data have also been used to study spring drought in central Himalaya [16], but remote sensing approaches to study drought in Nepal have been limited. Therefore, the use of satellite derived VCI to identify long-term spatial and temporal variation of drought in Nepal is crucial. Seasonal and timescale based studies are also valued for better drought investigation in Nepal. 

Long-term spatiotemporal assessment of drought in Nepal is important for societies and their environment. However, season and pixel based studies of drought on large temporal and spatial scales using remote sensing methods in Nepal remains uninvestigated. This study used VCI near infrared and red band data acquired from National Oceanic and Atmospheric Administration, Advanced Very High Resolution Radiometer (NOAA, AVHRR) sensors with long temporal series and moderate spatial resolution. This study aimed to find drought, trend and probability of drought on different time scales. Since the winter season of Nepal is dry and cold [22], vegetation is mostly dormant [50]. Therefore, we did not consider winter drought in this study. The pre-monsoon (VCI_3_: Mar to May), seasonal monsoon (VCI_6_: Jun to Nov) and annual (VCI_12_: Jan to Dec) represent short-term, medium-term and long-term drought for agricultural and hydrological implications in Nepal. The seasonal monsoon includes monsoon and post monsoon periods because 80% of the annual precipitation occurs during the monsoon and adequate moisture is stored in the soil during the post monsoon season [25]. The seasonal monsoon also represents the vegetation growing season. The relationship of VCI with temperature and precipitation has also been studied. The monitoring of drought using remote sensing to determine the probability of occurrence and relationships with climates on different time scales is important for improving agricultural production, protecting the environment and promoting sustainable socio-economic development [51]. This method of remote sensing of drought could also be a reference for developing environmental planning and implementing drought warning system and resistance measures in Nepal. 

## 2. Study Area and Data 

### 2.1. Study Area

**Figure 1:**
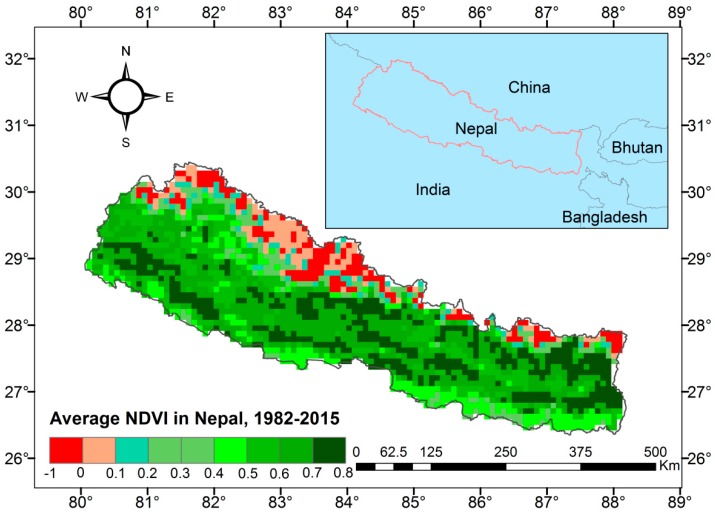
Location of Nepal and its average Normalized Difference Vegetation Index (NDVI) value from 1982–2015.

Nepal, located between 26°22′ and 30°27′ N latitude and 80°04′ and 88°12′ E longitude in the central part of the Himalayan region, is characterized by unique topography, climatic variations and vegetation distributions (Figure 1). The total area of the country is 147,181 km^2^ with 800 km parallel length from west to east and 200 km width from north to south. The altitude varies from 70 m in the south to 8848 m above sea level on Mount Everest in the north. The vegetation is categorized as forest (39.1%), cultivated lands (29.83%), shrub lands (3.40%) and grasslands (7.90%) [52]. The NDVI distributions shows more forests mainly located in the foothills of Nepal [25]. The annual precipitation ranges from 200 mm in parts of the northern Himalayan regions to 5500 mm in the south with a national average of 1800 mm and average temperatures can be as high as 30 °C in the south and as low as −10 °C in the north [22].

**Figure 2:**
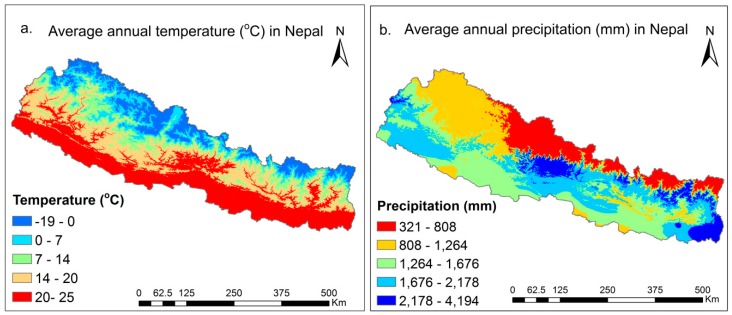
Climatic Research Unit (CRU) gridded downscaled to 1 km average annual temperature and precipitation distribution from 1982–2015 in Nepal.

Based on the Climatic Research Unit (CRU) monthly temperature and precipitation data obtained from the University of East Anglia [53], the average temperature and precipitation is high in southern parts of Nepal and decreased towards the north (Figure 2). The CRU data set is gridded to 0.5 × 0.5 degree resolution. The data were downscaled into the spatial resolution of 1 km using the Piecewise Cubic Hermite Interpolating Polynomial (PCHIP) method [54]. 

Figure 2 shows that an average annual temperature of Nepal is 13.71 °C with a maximum average of 25 °C in the south and a minimum of −19 °C in the north. Similarly, the average precipitation was 1428 mm with a maximum of 4194 mm in central and eastern Nepal and a minimum precipitation of 321 mm in the Trans-Himalaya and northern parts of Nepal. The spatial and temporal variations of the temperature and precipitation play an immense and important role for drought variations in Nepal. 

### 2.2. Data Acquisition

The VCI data were derived from the final NDVI data released by National Oceanic and Atmospheric Administration (NOAA) which are obtained from the Advanced Very High Resolution Radiometer (AVHRR) sensor spanning from 1982–2015. The NDVI data are the latest version of the data (NDVI3g.v1) with 15 days temporal and 8 km spatial resolution [55]. The monthly NDVI data were composited using the maximum value composite method [56]. The VCI based drought metric equation [57] was developed in to a global drought watch system using VCI derived from AVHRR NDVI data. The VCI was calculated by applying the following Equation (1) on the final NDVI data [42,58]:

(1) $$\mathrm{VCI}=100\times \frac{{\mathrm{NDVI}}_{i}-{\mathrm{NDVI}}_{\min}}{{\mathrm{NDVI}}_{\max}-{\mathrm{NDVI}}_{\min}},$$

where NDVI_i_ is the NDVI value of the pixel during a specific year i, and NDVI_max_ and NDVI_min_ are the maximum and minimum NDVI values, respectively, during a specific period from 1982–2015. The numerator is the difference between the actual and the minimum values of the NDVI and is indicative of the meteorology and vegetation information of a specific period. The maximum and minimum values of the denominator reflect the best and worst conditions of vegetative growth and the difference between them somewhat reflects the condition of the local vegetation [42,59,60]. The VCI contains both real-time and historical information of the NDVI. The VCI ranges between 0 and 100 in which smaller VCI values indicate worse vegetation growth and higher degrees of drought [42,59,60,61,62]. In addition, the monthly temperature data were collected from 40 meteorological stations and monthly precipitation data were collected from 174 meteorological stations across the country to analyze the relationship between VCI and climatic factors such as temperature and precipitation in Nepal. 

## 3. Methods

### 3.1. Identification of the Drought

The seasonal and annual VCI was derived from monthly NDVI data. The spatial and temporal variation of drought from 1982–2015 were characterized using drought grades defined by VCI. Based on the literature regarding aridity classification standards, droughts were divided into four grades; i.e., normal, slight drought, moderate drought and severe drought [3,43,46,47,58,63,64]. Here, we used three major types of drought grades based on VCI values developed for monitoring drought worldwide [59] as shown in Table 1.

### 3.2. Drought Trend Analysis

The linear regression method was used to analyze the annual and seasonal VCI trend in Nepal. The slope estimated by a regression model indicates the mean temporal change of VCI in which a positive slope indicates an increasing trend and a negative slope denotes a decreasing trend. An overall variation of the VCI trends in each pixel was calculated from 1982–2015 using Equation (2) [47,65]:

(2) $$\mathrm{Slope}=\frac{n\times \sum_{i=1}^{n}{\mathrm{VCI}}_{i}\times t_{i}-\left(\sum_{i=1}^{n}{\mathrm{VCI}}_{i}\right)\left(\sum_{i=1}^{n}t_{i}\right)}{n\times \sum_{i=1}^{n}t_{i}{}^{2}-{\left(\sum_{i=1}^{n}t_{i}\right)}^{2}},$$

where VCI_i_ is the vegetation condition index in year i, n is the length of the time series (n = 34) and t_i_ is an index number for the year 1982–2015 (1–34). VCI has a generally increasing trend if slope is greater than 0. An increasing trend for VCI reflects improved vegetation growth and drought abatement. 

### 3.3. Trend Detection of the VCI Drought Index

The Mann–Kendall (MK) test was applied to assess trends in time series of the VCI data [66,67]. This method has been widely used in various studies for trend detection due to its robustness [68]. The null hypothesis was tested at the 95% confidence level. The univariate MK statistics for time series data (Z_k_, k = 1, 2, …, n) was used as Equation (3):

(3) $$S=\sum_{j<1}^{n}{\mathrm{sgn}(Z}_{i}-Z_{j}),$$

where Z_i_ and Z_j_ are the mean VCI values in year i and j, respectively, i > j, n is the length of the time series and sgn (Z_i_ − Z_j_) is a sign function defined by Equation (4):

(4) $$\mathrm{sgn}\left(Z_{i}-Z_{j}\right)=\left\{\begin{array}{c} 1,\;\mathrm{if}\;Z_{i}-Z_{j}>1\\ 0,\;\mathrm{if}\;Z_{i}-Z_{j}=0\\ -1,\;\mathrm{if}\;Z_{i}-Z_{j}<0 \end{array}\right\}.$$

The null hypothesis (H_o_) is that there is no trend in the series, whereas the alternative hypothesis (H_1_) is that an increasing or decreasing monotonic trend exists in the series. The presence of a statistically significant trend was evaluated based on the *p*-value. H_o_ (no trend) is rejected if the *p*-value is less than a predefined significance level of 0.05. In addition to the linear regression method, Sen’s slope was also used to estimate the slope of VCI drought index. If the time series data presents a linear trend, the true slope (change per unit time) of a trend can be estimated by the non-parametric index developed by Sen [69], which is based on the assumption of a linear trend:

(5) $$\mathrm{Sen}’s\;\mathrm{slop}=\mathrm{Median}\left\{\left(x_{i}-x_{j}\right)/\left(i-j\right)\right\},\;i>j,$$

where x_i_ and x_j_ are the changing VCI values at time i and j, respectively. The slope of the annual and seasonal VCI trend is computed as an average change: a negative value indicates a negative trend and a positive value indicates a positive trend.

### 3.4. Anomaly Vegetation Condition Index 

The anomaly vegetation condition index (AVCI) was developed to analyze historical changes of the VCI and the level of soil moisture conditions that affects vegetation growth on annual and seasonal time scales. The AVCI was calculated using Equation (6) [64]:

(6) $$\mathrm{AVCI}=\frac{\left({\mathrm{VCI}}_{i}-{\mathrm{VCI}}_{\mathrm{ave}}\right)}{{\mathrm{VCI}}_{\mathrm{ave}}},$$

where VCI_i_ is the VCI value during a specific period, and VCI_ave_ is the average VCI value during the studied period from 1982–2015. A positive AVCI indicates that soil moisture is relatively abundant and better than average vegetation conditions, while a negative AVCI indicates moisture deficient soil and worse than average vegetation conditions.

### 3.5. Exceedance Probability and Return Periods 

The exceedance probability and return periods of the VCI were computed using Weibull’s frequency distribution equation [70]. The exceedance probability and return period are reciprocal to each other as follows as in Equation (7):

(7) $$T_{r}=\frac{n+1}{m}=\frac{1}{p(\mathrm{xm})},$$

where p(xm) denotes the exceedance probability, T_r_ is the return period that indicates an average number of years within which a given event will be equaled or exceeded, n is the total number of the study period (34 years) and m is the rank of the observations in descending order 

### 3.6. Correlation Analysis of the VCI and Climate Factors

The Pearson correlation coefficient (*r*) between the VCI and climatic factors (i.e., temperature and precipitation) on the annual, seasonal monsoon and pre-monsoon time scales was calculated to assess the relationship between drought and climatic factors [46]. The Pearson correlation analysis was conducted using the statistics package in R and the *t*-test [65] was performed for trend analysis significance. If the correlation value between two variables is positive and the *p*-value is less than 0.05, it is believed that correlation is statistically significant. 

## 4. Results 

### 4.1. Temporal Variation of VCI 

**Figure 3:**
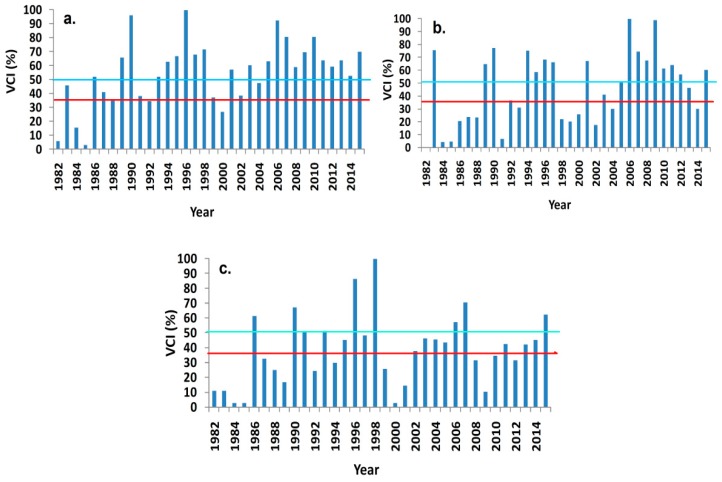
Temporal variation of the drought index (VCI) in different time scales; (**a**) annual VCI (VCI_12_); (**b**) seasonal monsoon VCI (VCI_6_) and (**c**) pre-monsoon VCI (VCI_3_) from 1982–2015 in Nepal.

The spatially averaged VCI on the annual, seasonal monsoon and pre-monsoon scales in Nepal were 55.12%, 46.28% and 38.45%, respectively. The annual VCI has increased at the rate of 1.14 yr^−1^. Similarly, the VCI during the seasonal monsoon and pre-monsoon season also increased at the rate of 1.31 yr^−1^ and 0.77 yr^−1^, respectively. From 1982–1990, the VCI increased on the annual, seasonal monsoon and pre-monsoon scales at the rate of 8.30 yr^−1^, 5.56 yr^−1^ and 5.26 yr^−1^, respectively. From 1991–2000, the annual VCI was also slightly increased, but the seasonal monsoon and pre-monsoon VCI decreased by 0.03 yr^−1^ and 0.47 yr^−1^, respectively, which implied an increased tendency of drought. Again from 2001 to 2015, VCI showed slight increases on all three timescales. The long-term annual drought analysis (VCI_12_) indicated that severe drought was identified in 1982, 1984, 1985 and 2000 while normal drought years were 1983, 1987, 1988, 1991, 1992, 1999, 2002 and 2004. In the monsoon season, severe droughts mainly occurred before 2004 and the normal drought was found in 1992, 2001, 2003 and 2013. Most droughts occurred during the pre-monsoon season than during monsoon and post monsoon season. In general, VCI improved after 2005, but there were several drought years during the pre-monsoon season in Nepal. The inter-annual variation of the VCI drought index in the long term (annual), medium term (seasonal monsoon) and short term (pre-monsoon) is given in Figure 3. The lines parallel to the *x*-axis in Figure 3 represent threshold values of VCI for severe drought and drought. 

The red line indicates the threshold of severe drought (VCI ≤ 35%) and the blue line indicates the threshold of drought (35% ≤ VCI ≤ 50%). During the pre-monsoon season, more severe drought years were identified than drought years. However, higher VCI (>70%) was also reported in 1996 and 1998 during the pre-monsoon season. In the monsoon season, the number of drought years was less than that in pre-monsoon mainly found before 2005. After 2005, VCI was normally greater than 35% except for that in 2014. The highest VCI was found in 2006 and 2009. On the annual time scale, no severe drought (≤35%) was identified as all VCI values were larger than 35%. On the seasonal monsoon time scale, severe drought was identified in 2014. In the pre-monsoon season, severe drought has identified in 2008, 2009 and 2012. During 1982–2015, the number of severe drought years on the annual time scale was 4, on the seasonal monsoon scales was 14 and on the pre-monsoon time scale was 16 as shown in Figure 3. Overall, the annual average VCI trend during all three time scales from 1982 to 2015 has increased which reflects improved vegetation and less risk of drought.

### 4.2. Spatial Variation of VCI 

**Figure 4:**
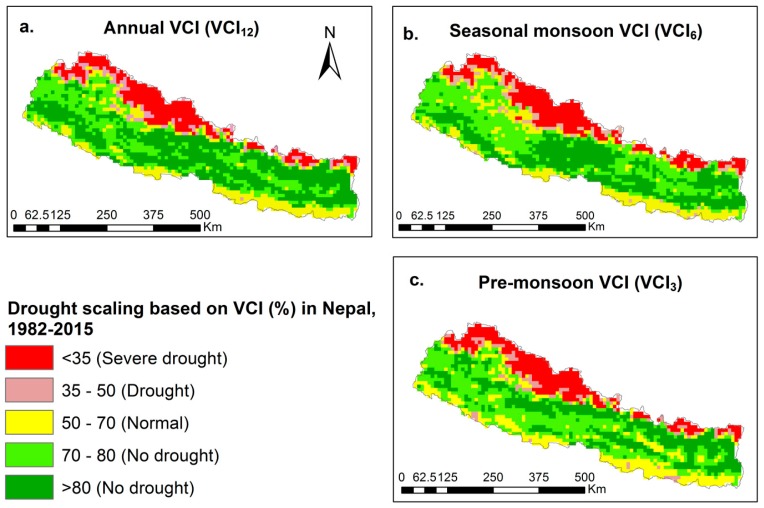
Spatial variation of the temporally averaged VCI for different time scales; (**a**) annual VCI (VCI_12_); (**b**) seasonal monsoon VCI (VCI_6_) and (**c**) pre-monsoon VCI (VCI_3_) from 1982–2015 in Nepal.

The temporally averaged VCI was mostly greater than 70% VCI in the foothills, lower than 50% VCI in the northern regions and slight to moderate i.e., average between 35% to 50% VCI in the southern plains due to the variations of altitude and climate (Figure 4). The temporally average VCI identified drought during the seasonal monsoon and pre-monsoon season, but the average VCI was greater than 50% on the annual time scale. On the annual time scale, the temporally averaged VCI was severely low i.e., less than 35% in the northwestern regions (Figure 4a). This area lies in the rain shadow zone where precipitation trends are also very low compared to other places in Nepal. In the southern parts, the temporally averaged VCI ranged from 50% to 70% which indicated almost no drought on all time scales, but the VCI in southern parts was lower compared to that in foothills. The hilly regions, especially in the central and eastern foothills, experienced wet condition where the VCI was greater than 70%.

On the seasonal monsoon scale (Figure 4b), the VCI was higher in the central and eastern hills. In the western regions, the temporally averaged VCI in seasonal monsoon scale was less than that on the annual time scale. In the pre-monsoon season (Figure 4c), more severe droughts were identified in the northwestern regions. The plain areas mostly covered by agricultural land predicted normal temporally averaged VCI ranging from 50% to 70%. The average VCI was lower in hilly areas during the pre-monsoon than the seasonal monsoon and the annual time scales. During the pre-monsoon period, some parts of western and eastern Terai (southern Nepal) were identified as being in drought (Figure 4c). The upper hills areas in the western regions were identified as being in drought with the temporally averaged VCI ranging between 35–50%. 

### 4.3. Spatial and Temporal Drought Trends

**Figure 5:**
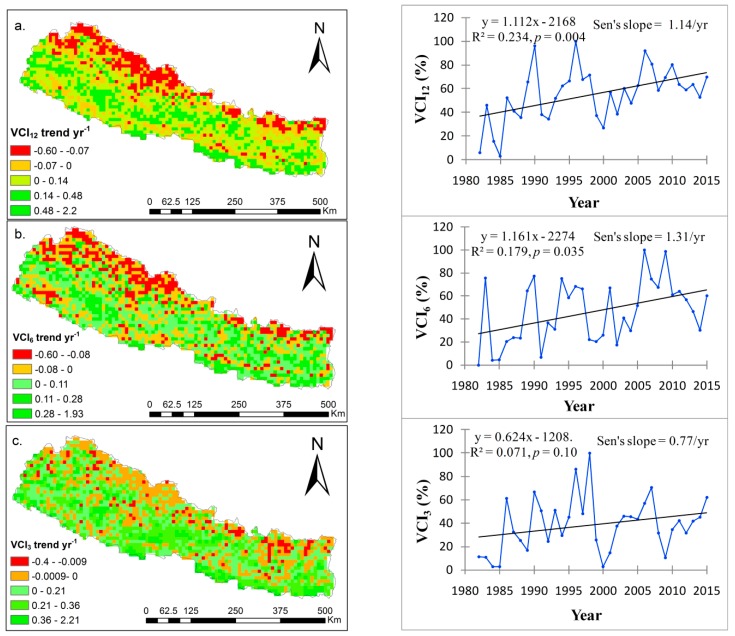
Spatial and temporal trend of VCI; (**a**) annual (VCI _12_); (**b**) seasonal monsoon (VCI _6_) and (**c**) pre-monsoon (VCI_3_) VCI trends in Nepal from 1982–2015.

The spatial drought trend based on a linear slope of each grid point in the map and the temporal trend based on the Mann–Kendall test and Sen’s slope are shown on the annual (Figure 5a), seasonal monsoon (Figure 5b) and pre-monsoon scales (Figure 5c). The overall annual VCI trend (VCI_12_) has significantly increased at the rate of 1.14 yr^−1^ (*p* = 0.004) with a higher positive trend after 2000. This increased VCI trends implies decreased drought. The VCI trend is spatially varied and mostly negative in the northern area. The greater negative VCI trends in the western and far western regions indicate potential drought and drought vulnerability. In some parts of the eastern mountains and southern plains, the VCI decreased at the rate of 0.07 to 0.6 yr^−1^ and 0 to 0.07 yr^−1^, respectively, which indicate increased risk of drought. On the seasonal monsoon scale (Figure 5b), the VCI largely decreased in the western mountains, far-western mountains, foothills and some parts of Terai (lowlands), also indicating risk of drought. Therefore, these regions are identified as being more vulnerable towards drought in Nepal.

On the seasonal monsoon scale, the VCI has significantly increased at the rate of 1.31 yr^−1^ (*p* = 0.03), which was similar to the annual VCI trend (Figure 5b). During the pre-monsoon season, VCI also increased at the rate of 0.77 yr^−1^ (*p* = 0.1) indicating decreased drought. However, the decreased VCI in the foothills and mountains indicate increased drought (Figure 5c). The spatial distribution results show that a negative VCI trend of 0.4 yr^−1^ was observed in the eastern hills, central mountains and the western and far western regions, which imply increasing trends of drought. The inner southern parts of the plains area which is mostly occupied by agricultural lands identified slight drought trends (Figure 5a–c) on all investigated time scales. 

### 4.4. Anomaly Vegetation Condition Index 

**Figure 6:**
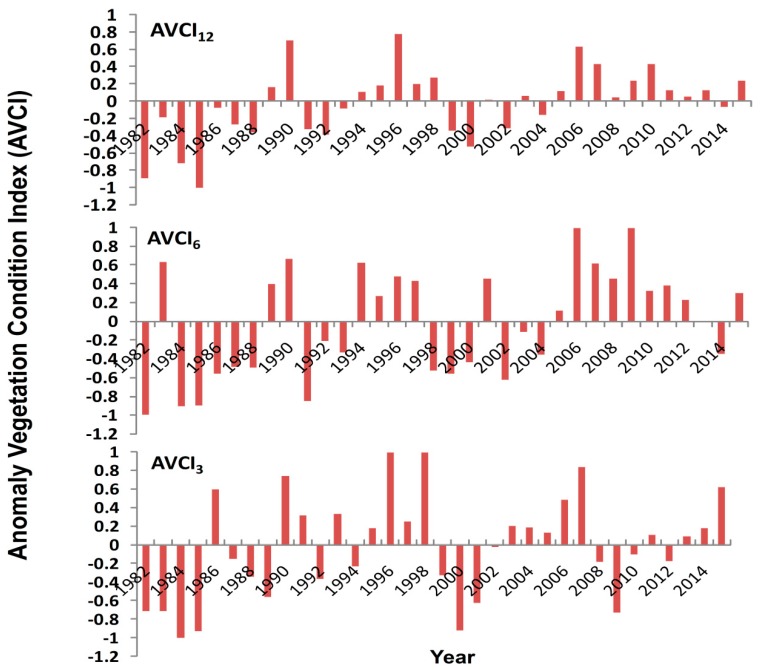
Anomaly of the vegetation condition index for annual (AVCI_12_), seasonal monsoon (AVCI_6_) and pre-monsoonal (AVCI_3_) season from 1982–2015 in Nepal.

The overall temporal variation in the AVCI trends on the annual, monsoonal and pre-monsoon time scales were positively increased at the rate of 0.019 yr^−1^, 0.025 yr^−1^ and 0.016 yr^−1^, respectively (Figure 6). This indicates abundant moisture conditions than required for vegetation growth. However, inter-annual variation of AVCI fluctuated. The annual AVCI indicated moisture deficiencies in several years. The maximum negative AVCI reached –1 in 1986 followed by the next most negative years in 1984, 1982 and 2000. The maximum positive AVCI of 0.6 was observed in 1996 followed by the next most positive year in 1990 and 2006.

After 2005, the AVCI condition was positive, indicating that the soil moisture condition was relatively abundant and higher than required for average vegetation conditions. In the beginning of the study period, the soil moisture was relatively more negative, especially in 1982, 1984, 1985, 2000, 2002 and 2014 on all time scales. In the monsoon season, a higher positive AVCI was observed in 1983, 1990, 1994, 2006 and 2009. In the pre-monsoon season, the AVCI was negative for several multi-year periods with the highest negative values in 1984, 2001 and 2009, indicating moisture deficiencies. The AVCI was highly positive in 1990, 1996, 1998 and 2007. Overall, the soil moisture condition indicated by AVCI was relatively adequate during the monsoon season compared with the annual average and pre-monsoon season. Overall, the analysis showed positive increases in the spatially averaged AVCI but with high inter annual variation on all time scales. 

### 4.5. Exceedence Probability and Return Periods

**Figure 7:**
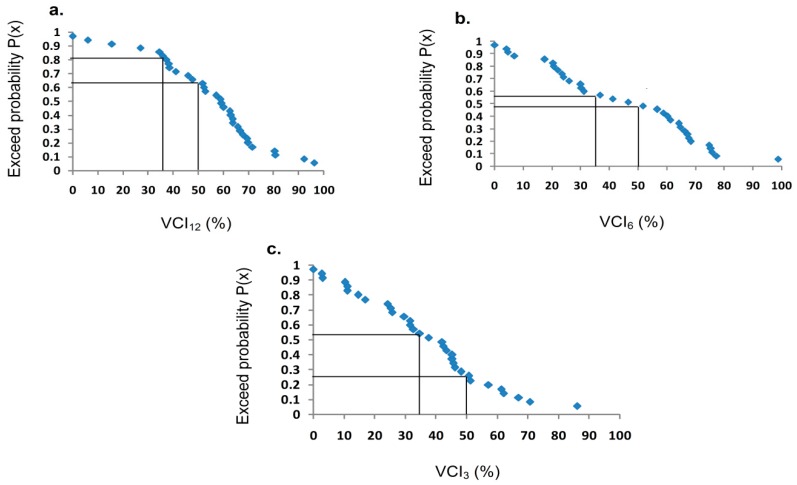
Drought index probability curve: (**a**) annual; (**b**) seasonal monsoon and (**c**) pre-monsoon from 1982–2015 in Nepal.

Figure 7 shows the exceedance probability of Spatially Averaged Nepalese VCI equal to or greater than 35% and 50% on annual, seasonal monsoon and pre-monsoon scales from 1982–2015 in Nepal. The annual VCI equal to or greater than 35% and 50% corresponded to an exceedance probability of 0.80 and 0.65, respectively. These results predict that there was a 20% chance of severe drought occurring (VCI < 35%) and a 35% chance of normal drought occurring (35% ≤ VCI ≤ 50%) in Nepal. On the seasonal monsoon and pre-monsoon scales, the exceedance probability for spatially averaged VCI equal to or larger than 35% was 0.55, which predicts that the probability of severe drought occurring was 45% on these time scales in Nepal. 

The return period of the annual spatially averaged VCI equal to or more than 35% and 50% corresponded to 1.25 and 1.53 years, respectively. It was relatively greater for seasonal monsoon scales with a return period 1.81 and 2.22 years, respectively. The return period of VCI less than 35% (severe drought) was five years on the annual scale and 2.22 years on seasonal monsoon and pre-monsoon scales. However, the return period of normal drought (35% ≤ VCI ≤ 50%) during the pre-monsoon season was four years, which was less frequent than the annual and seasonal monsoon scales. 

### 4.6. Relation between VCI and Climatic Factors from 1982–2015 in Nepal

The Pearson correlation between the spatial averaged VCI and temperature was positive and significant on the annual (r = 0.41, *p* = 0.01) and seasonal monsoon scales (r = 0.36, *p* = 0.03) but negative for the pre-monsoon season. The correlation between spatially averaged VCI and precipitation was positive on the pre-monsoon scale and negative on the annual and seasonal monsoon scales (Table 2). These results indicate that temperature was the main influencing factor on spatially averaged VCI during the annual and seasonal monsoon periods. 

The significant positive correlation between spatially averaged VCI and temperature indicated that an increase in mean temperature caused an upward trend in the VCI, which implies a decline in drought. The negative correlation between spatially averaged VCI and precipitation might have caused the drought to have a delayed response to the precipitation, where the time lag effect was not considered in this study. Many studies indicate that VCI reacts with delay to the change of moisture conditions and this reaction is controlled by the previously accumulated soil water storage [71]. The positive correlation of precipitation with spatially averaged VCI in the pre-monsoon season implies that increased precipitation supports vegetation growth and vice versa. 

## 5. Discussion

In this study, we explored long-term drought trends based on the spatially averaged VCI index for annual (VCI_12_: Jan–Dec), seasonal monsoon (VCI_6_: June–Nov) and pre-monsoon (VCI_3_: March–May) time scales. The seasonal monsoon is generally considered as a wet season that includes monsoon and post monsoon seasons. The monsoon season of Nepal covers June, July, August and September when more than 80% of monsoons occurred and the post monsoon season covers October and November. The intense monsoons during the June and July months have large moisture influences on the post monsoon season. Similarly, the retrieval of high NDVI during autumn and the lengthening of the growing season [25] suggest that the seasonal monsoon period from June to November would be appropriate to identify medium-term drought based on vegetation. Therefore, both monsoon and post monsoon seasons were considered as a seasonal monsoon period for drought identification. The discussions are focused on the identification and spatiotemporal variation of VCI, the probability of severe drought and its relationship with climate parameters such as temperature and precipitation from 1982–2015 in Nepal.

### 5.1. Identification and Temporal Variations of VCI from 1982–2015

Temporally, the spatially averaged VCI was greater than 50% on the annual time scale, but it was less than 50% for the seasonal monsoon and pre-monsoon seasons. The overall temporal trend of the VCI drought index was positive with an inter-decadal variation in Nepal. The spatially averaged VCI was significantly increased in annual and seasonal monsoon periods but did not significantly changed during the pre-monsoon season. This positive tendency of the spatially averaged VCI indicates a decreasing trend of drought. The VCI trend was highly increased at the beginning of the study period, but then decreased from 1990 to 2000 in the pre-monsoon and monsoon seasons, indicating an increased tendency for drought. The severe drought has been predicted in several years on pre-monsoon and seasonal monsoon scales. In this study, three averaging time scales, i.e., annual, pre-monsoon and seasonal monsoon, were used to investigate remote sensing of agriculture and hydrological drought. During our study period, several drought years have been observed and the pre-monsoon drought were consistent with the drought found using tree ring growth in Nepal [16]. This study also determined the inter-annual fluctuation of soil moisture based on AVCI (Figure 6). The overall trends of AVCI were positive on all time scales, but there were several moisture deficient years during the pre-monsoon and seasonal monsoon periods. The spatially averaged AVCI also reached as low as −1 in some of the years, which indicates moisture deficiency and a drought year in Nepal. Comparing Figure 3 and Figure 6, all severe drought years during the annual, seasonal monsoon and pre-monsoon periods identified moisture deficiencies i.e., negative AVCI. From 1990−2000, spatially averaged AVCI trends were negative during the pre-monsoon and seasonal monsoon scales. After the year 2000, spatially averaged AVCI trends predicted improved soil moisture conditions. Soil moisture does not always depend on intensity and duration of the rainfall because a large intensity of rain water flows down to the rivers instead of being retained as moisture in Nepal [72]. Therefore, the use of AVCI can give the soil moisture condition deviated from optimum requirements for vegetation growth because soil moisture is also a dominant factor for vegetation changes in the Trans-Himalayan regions and far western regions in Nepal [73,74]. It also played a primary role in South Asian vegetation changes, especially in dry regions [75]. 

### 5.2. Spatial Variation of the VCI from 1982–2015

The high topographic variation of Nepal possesses a diverse bio-physical environment from the south to north so that temperatures, precipitation, wind velocity, humidity and sunshine hours were different. Consequently, the VCI values are spatially varied. The northwestern parts mainly in the Trans-Himalayan regions indicated severe drought, while the lowlands, i.e., Terai, indicated normal VCI. However, the VCI predicted that the foothills experienced hardly few droughts in Nepal. The northwestern mountains indicate more severe drought on all time scales, which makes sense based on the fact that it lies on the leeward side, where the average rainfall was very low [22] and moisture was the limiting factor for plant growth [74]. For instance, Mustang, one of the districts in the northwestern regions only received 76.63 mm average annual rainfall during the last 30 years. The snowfall rate, the main source of water, also decreased. In turn, severe water scarcity occurred [76] in this region. The study supports the conclusion that moisture supply is a primary factor limiting vegetation activities in dry regions [75]. In these dry regions, soil moisture and plant evapo-transpiration are scarce forming rather arid, semi-desertic zones with low precipitation and scarce vegetation. The low evapo-transpiration in turn lessens the atmospheric relative humidity, less relative humidity results in less probability of rainfall, as it became harder to reach saturation conditions and thereby increased drought. Therefore, the areas that were drought prone areas and that experienced prolonged drought were lands occupied only by grasses and shrubs. However, the southern parts of Nepal received more rain than the northern parts [21] because of high orographic variation: the winds containing rain coming from the Bay of Bengal then first stroke the mountains and returned back to the southern parts. These differences in the precipitation distribution also have influences on the vegetation. 

The northern parts possessed low lying alpine vegetation with montane grasslands and shrub lands reflecting low VCI values. The low VCI values were observed mainly in the Trans-Himalayan regions in northern Nepal where the moisture limited plant growth. Conversely, the foothills possessed temperate broadleaved and sub alpine coniferous forest that results in larger VCI values. The drought condition predicted by VCI in the far western regions was higher on all time scales, which makes sense because this region is located far from the ocean where it is hard to receive rain containing winds. During the monsoon season, central and eastern Nepal had larger VCI values which indicated fewer droughts. For example, Lumle in central Nepal had average annual precipitation of more than 5000 mm [25]. For the pre-monsoon season, VCI values also decreased in eastern hills and mountain areas which implies increasing drought. The lower VCI values as shown in Figure 4 and the lower VCI trends depicted in Figure 5 potentially indicates less plant evapo-transpiration, reducing humidity and rainfall, and which may increase the risk of soil moisture deficiency and consequent drought. The VCI values were the normal in lowlands which may be due to agricultural practices, as the lowlands are the most cultivated region in Nepal [77]. However, high population [78], deforestation, sedimentation, land conversion from forest to agriculture and construction may have led to the observed decline in VCI in Terai (the low lands). The identification of drought based on VCI and spatially averaged VCI trends in fact has positive implications on local agriculture activities. 

### 5.3. Probability of Occurring Severe Drought and Drought Based on the VCI Index 

In this study, we analyzed the exceedance probability and return periods of spatially averaged VCI from 1982–2015 on the annual, seasonal and pre-monsoon scales. The exceedance probability represents the average number of years within which a given event will be equaled or exceeded. These parameters are reciprocal with each other as both parameters were computed in the order of descending with a rank of 1 to the highest value, a rank of 2 to the next highest value and so on. Weibul’s formula was used to avoid the difficulties of having a finite sample (m = n) and which is a commonly used in studies of hydrology and the environment. The exceedance probability analysis result predicted that probability of occurring severe drought was 20% and occurring normal drought was 35% on the annual time scales. Similarly, the probability of occurring severe drought was 45% on both the seasonal monsoon and pre-monsoon scales. It seems high, but return periods are low, i.e., 5 years on the annual and 2.22 years on the pre-monsoon and seasonal monsoon scales. The predicted return periods and exceedance probability of the VCI larger than 35% to 50% were relatively higher than VCI values less than 35%. However, the probability and return periods of VCI ≥ 50% were frequent and higher in Nepal. The positive trend of VCI from 1982–2015, less probability of occurring severe drought and return periods implies less drought, which agrees with an increasing trend of NDVI at the rate of 0.0008 yr^−1^ [25] and of forest coverage increasing from 39.1% in 2010 [52] to 40.36% in 2015 [79]. The NDVI in 83.89% (57.35% significant) areas found increasing trends and 16.10% (4.68% significant) of the areas present the decreasing trends, and the significant changes of NDVI in the pre-monsoon and monsoon season are 38.88% and 43.46%, respectively [25]. The high variability of this vegetation could have influence on the variability in probability and return periods of the VCI based drought. However, the drought should be studied in detail using a variety of drought indices, remote sensing techniques and in situ data for further prediction in Nepal. 

### 5.4. Relationship between VCI and Climates

The precipitation and temperature played a crucial role in the VCI variations at different locations and time periods. There are several types of drought definition i.e., meteorological drought (dry weather), hydrological drought (low water supply), agricultural drought (declining soil moisture) and socioeconomic drought (failure of water resources). These droughts cannot be detected by the VCI until they impact vegetation cover. Therefore, identification of the relationship between vegetation based drought and the climate is of utmost. The significant positive correlation between spatially averaged VCI and temperature on annual and seasonal monsoon scales indicates that temperature is an important factor for drought. The increased temperature may lengthen the growing season and photosynthesis until its optimum level. The average temperature during the annual and seasonal monsoon periods was 19.47 °C and 22.26 °C in which the spatially averaged VCI value was 55.12% and 46.28%, respectively, both of the temperature and VCI had positive trends from 1982–2015. On the pre-monsoon scale, the spatially averaged VCI had negative correlation with temperature. In Nepal, the pre-monsoon precipitation trend was significantly negative, but total rainfall during the pre-monsoon season represented only 7.96% of annual rainfall [26]. The spatially average VCI and precipitation for pre-monsoon season was 38.45% and 222.63 mm, respectively. Pre-monsoon precipitation also decreased at the rate of 0.454 mm·yr^−1^ from 1982–2015. The decreased trend of the pre-monsoon precipitation may decrease the VCI and consequently increase drought. Both of these low average precipitation and spatially averaged VCI in the pre-monsoon season can indicate drought. 

The studies showed that NDVI was positively correlated with the temperature in the northern mid and high latitudinal zones [80], the Northern Hemisphere, China and the Tibetan Plateau [81]. The temperature is the primary driver for 64% variations of the global vegetation growth from 1982–2008 [82]. In Nepal, NDVI is positive and significantly correlated with the temperature but negatively correlated with the precipitation from 1982–2015 [25]. Furthermore, positive NDVI trends were also attributed to CO_2_ in Nepal [83]. In this study, VCI was also positive and significantly correlated with temperature. The VCI contains both real-time and historical information of the NDVI [47]. As Nepal is a mountainous country, high temperature may reduce the snowfall rate (source of water) resulting in severe water scarcity and hard to reach soil saturation conditions [76]. The low soil saturation condition (i.e., dry soil) lessens the plant’s evapo-transpiration rate and consequent low atmospheric humidity that reflects less probability of rainfall occurring. Therefore, temperature can be a primary driver for VCI and VCI based drought indication in the mountainous country like Nepal. The negative correlation between VCI and precipitation might have caused the vegetation to have a delayed response to the drought. Many studies indicate that VCI reacts with delay to the change of moisture conditions and this reaction is controlled by the previously accumulated soil water storage [71]. In monsoons, the soil is saturated with melting snow water and excess moisture can worsen vegetation conditions. Agricultural activity in the arable land usually starts when soil becomes rather dry [84]. Other studies also show that forests respond to drought on the long-term scales, while arable land on the short-term scales [85]. Using the same GIMMS NDVI3g data from 1982–2008, global vegetation studies showed time lag effects [82]. Therefore, Nepal may also have a time lag effects on vegetation related to precipitation. The high mean NDVI during the post monsoon (October and November) season [25] and high rainfall during the monsoon season (June, July, August, and September) [22] also justify that some time lag effects exist. Thus, delayed response of VCI to precipitation might be a reason to have a negative correlation between the VCI and precipitation in Nepal. The strongest connection between precipitation and VCI was also determined in the areas with low soil water–holding capacity [86]. Aside from temperature and precipitation, drought was also connected with a broad scale climatic variability. Summer drought has been linked with the Southern Oscillation Index (SOI) and the Indian Ocean Dipole Mode Index (DMI) [12]. Drought was also linked with the El Nino Southern Oscillation and the Atlantic Multi-decadal Oscillation (AMO) in the Trans-Himalayan region [17] of Central Himalaya in Nepal.

### 5.5. Uncertainty, Validation and Applicability of VCI-Based Drought Study

The use of remote sensing data and choice of the vegetation index may have some uncertainties due to its coarse resolution. The sensors, data acquisition, calibrated procedures and grid data interpolation may have remain some uncertainties. The spatially averaged VCI also have some limitation, though it covers the average of both NDVI maximum and NDVI minimum values during its retrieval process. However, this data set has been widely used to identify and prediction of the drought globally [3,42,43,46,47,57,58,59,61,63,64] because of high quality and eliminates possible errors by both intraneous (e.g., calibration, view geometry, volcanic aerosols, solar zenith angle, orbital drift, and sensor inconsistency) and extraneous factors [87,88,89,90]. The study of drought for all of Nepal is limited. This study is the first on drought based on a long-term time series satellite based VCI. Some previous studies were conducted, but all of them are regional, seasonal, site specific and short temporal ranges. The baseline drought data on the annual and seasonal scales at the country level are not available to compare with our results. However, some results of the previous study and precipitation records were used to support our findings. The Table 3 listed the number of years having VCI ≤ 35% (considered as severe drought) and VCI 35% ≤ VCI ≤ 50% (considered as the normal drought).

In our study, 4, 14 and 16 years are severe drought years and 8, 4 and 11 years are normal drought years from 1982–2015 on the annual, seasonal monsoon and pre-monsoon time scales. Previous studies showed that Nepal experienced drought in 1982, 1986, 1992, 1994, 1997, 2002, 2008, 2009, 2012, 2013 and 2015 and also frequent dry spells from 2002 to 2006 during both the dry and wet monsoons [11]. Droughts over the last 33 years are moderate to extreme intensity in Nepal [12]. Drought often occurred in the mid and far western parts of Nepal [13], especially during the summer season [14]. The worst widespread droughts in the central Nepal were recorded in 2004, 2005, 2006, and 2009 in the summer [15]. Our results predicted drought mostly in the mid and far western regions where spatially averaged VCI values are relatively less. The 405 years of long tree ring chronology showed that the Trans Himalayan regions experienced drought [17]. In our results, the Trans-Himalayan regions also experienced severe drought where spatially averaged VCI values from 1982–2015 was less than 35% and decreased on all of the time scales (Figure 4 and Figure 5). Similarly, spring drought has also been predicted using tree ring chronology in the Central Himalaya [16], which supports our less obtained spatially averaged VCI from 1982–2015 in the pre-monsoon season i.e., 38.45%. To verify our results, we have measured the accuracy test between meteorological drought and VCI based drought in severe and drought years during pre-monsoon seasons because the spatially average VCI is lower than 50% and more drought years were found in pre-monsoon seasons. The accuracy of the predicted severe drought in the pre-monsoon season is 62.5%, in which 10 out of 16 severe drought years have lower rainfall than average pre-monsoon rainfall from 1982–2015. Similarly, the accuracy of the predicted normal drought years is 72.72% in which 8 out of 11 normal drought years have lower rainfall than average pre-monsoon rainfall from 1982–2015. The average pre-monsoon rainfall from 1982–2015 is 222.63 mm. It showed that meteorological droughts based on precipitation are somehow similar with VCI based drought in the pre-monsoon season in which the accuracy is up to 73%. However, meteorological drought may not be directly detected by VCI before it influences vegetation growth.

In this vacuum of drought study for all of Nepal, this study can provide baseline drought information in Nepal. It also seeks the relevancy of remote sensing applications for drought studies at the global, regional and country levels. The multiple satellite based indices with continuous and long time series records obtained from high resolution sensors can be used for better prediction of droughts. Aside from only the NOAA’s AVHRR NDVI data, the MODIS 16-day composite NDVI dataset with 250 m spatial resolution for 2000–2016, Satellite Pour l’ Observation de la Terre (SPOT) 10 days composited NDVI data with 1 km resolution for 1998–2014 and Landsat images of 30 m resolution can be used to retrieve VCI and droughts in future. 

## 6. Conclusions

This study investigated spatial and temporal variation of droughts based on satellite derived VCI and the relationship with the climate in the past three decades from 1982 to 2015 in Nepal. The results identified that severe drought was identified in 1982, 1984, 1985 and 2000 on all time scales. Severe drought years were identified more on the seasonal monsoon and pre-monsoon time scales. However, the VCI has increased at the rate of 1.14 yr^−1^ (*p* = 0.04), 1.31 yr^−1^ (*p* = 0.03) and 0.77 yr^−1^ (*p* = 0.77) on the annual, seasonal monsoon and pre-monsoon time scales, respectively. These positive trends of VCI imply decreased drought despite the fact that drought had inter-annual and spatial variation. Spatially, severe drought was identified in the Trans-Himalayan regions located in the northwestern parts of Nepal. The results predicted increasing drought in the west and far-western regions and a few parts of the eastern foothills and mountains during the pre-monsoon seasons. Soil moisture deficiency, i.e., negative AVCI, was identified before 2000. On the seasonal scales, soil moisture was relatively abundant during the monsoon season compared with the annual and pre-monsoon season. On annual time scales, VCI predicted probability of occurring severe drought was 20% and of normal drought occurring was 35%. However, the chance of occurring severe drought was 45% on the seasonal monsoon and pre-monsoon scales. These changes of VCI were also linked with climatic parameters in which the temperature on annual and seasonal monsoon scales and precipitation in the pre-monsoon season were positively correlated. The novelty of this study lies in the fact that it provides a first assessment of the drought based on satellite derived VCI over all of Nepal. In order to improve the identification and prediction of drought, the detailed study could be conducted using remote sensing data of multiple sources and a variety of drought indices in Nepal. 

## Figures and Tables

**Table 1:** Drought grades defined by Vegetation Condition Index (VCI).

Grade	Types	VCI (%)
1	Normal	>50
2	Drought	35–50
3	Severe drought	<35

**Table 2:** Correlation between spatially averaged VCI and climate (i.e., temperature and precipitation) from 1982–2015; R_t_: VCI and temperature and R_p_: VCI and precipitation.

Time Scale	R_t_	*p*-Value	R_p_	*p*-Value
Annual	0.41	0.01	−0.088	0.62
Seasonal Monsoon	0.36	0.03	−0.22	0.2
Pre-monsoon	−0.06	0.70	0.06	0.71

**Table 3:** Spatially averaged VCI, severe drought years and normal drought years in the annual, seasonal monsoon and pre-monsoon time scales from 1982–2015.

Drought Scales	VCI (%)	Severe Drought Years (VCI < 35%)	Drought Years (35% ≤ VCI ≤ 50%)
Annual (VCI_12_)	55.12%	1982, 1984, 1985, 2000	1983,1987, 1988, 1991, 1992, 1999, 2002, 2004
Seasonal Monsoon (VCI_6_)	46.28%	1982, 1984, 1985, 1986, 1987, 1988, 1991, 1993, 1998, 1999, 2000, 2002, 2004, 2014	1992, 2003, 2005, 2013
Pre-monsoon (VCI_3_)	38.45%	1982, 1983, 1984, 1985, 1987, 1988, 1989, 1992, 1994, 1999, 2000, 2001, 2008, 2009, 2010, 2012	1991,1993,1995,1997,2002, 2003,2004,2005,2011,2013,2014
